# Maternal Relationships among Ancient and Modern Southern African Sheep: Newly Discovered Mitochondrial Haplogroups

**DOI:** 10.3390/biology11030428

**Published:** 2022-03-11

**Authors:** K. Ann Horsburgh, Devri B. Beckett, Anna L. Gosling

**Affiliations:** 1 Department of Anthropology, Southern Methodist University, Dallas, TX 75275, USA; 2 School of Geography, Archaeology and Environmental Studies, University of the Witwatersrand, Wits 2050, South Africa; 3 Department of Anthropology, University of Colorado, Denver, CO 80217, USA; devri.beckett@ucdenver.edu; 4 Department of Anatomy, University of Otago, Dunedin 9016, New Zealand; anna.gosling@otago.ac.nz

**Keywords:** sheep, mitochondrial genome, ancient DNA, southern Africa, archaeology, Later Stone Age

## Abstract

**Simple Summary:**

The genetic diversity of southern African sheep remains under-studied. We present here the complete mitochondrial genomes of archaeological southern African sheep, as well as the genomes from three indigenous southern African breeds—Damara, Namaqua Afrikaner, and Ronderib Afrikaner. We show that southern African sheep exhibit limited genetic diversity which is consistent with our understanding of their migration south from northernmost Africa. Intriguingly, many of the modern sheep show close relationships with the archaeological sheep, implying an ancestor-descendant relationship. Similarly, the sheep that do not exhibit a close relationship with the archaeological sheep nonetheless cluster closely with each other and do not show a close relationship with European and Asian sheep. This suggests that they too are descendants of indigenous sheep and not the product of historic introductions of exotic breeds.

**Abstract:**

We investigated the genetic diversity and historic relationships among southern African sheep as well as the relationships between them and sheep outside the continent by sourcing both archaeological and modern sheep samples. Archaeological sheep samples derived from the site Die Kelders 1, near Cape Town, date to approximately 1500 years ago. The modern samples were taken as ear snips from Damara, Namaqua Afrikaner, and Ronderib Afrikaner sheep on a farm in Prieska in the Northern Cape. Illumina sequencing libraries were constructed for both ancient and modern specimens. Ancient specimens were enriched for the mitochondrial genome using an in-solution hybridization protocol and modern specimens were subjected to shotgun sequencing. Sequences were mapped to the *Ovis aries* reference genome, assigned to haplogroups and subhaplogroups, and used to calculate a phylogenetic tree using previously published, geographically dispersed mitochondrial genome sheep sequences. Genetic diversity statistics show that southern African sheep have lower diversity than sheep in other regions. Phylogenetic analysis reveals that many modern southern African sheep are likely descended from prehistoric indigenous sheep populations and not from sheep imported from Europe during the historic period.

## 1. Introduction

More than 400 million sheep (*Ovis aries*) representing approximately 170 breeds live in Africa [1], but they are not indigenous to the continent. They were likely domesticated from the European mouflon (*Ovis aries musimon*) and the Asiatic mouflon (*Ovis orientalis*) in southwestern Asia about 10,000 years ago [2,3,4,5,6]. Northern Africa is home to the Barbary sheep (*Ammotragus lervia*, and not technically a sheep but a caprine), however, despite archaeological evidence that they were likely confined and even fed sedating fodder [7,8,9,10], there is no evidence that they were ever domesticated, nor evidence that they interbred with domestic sheep breeds [11]. The oldest evidence of domestic sheep in Africa comes from the 5th millennium BC from Haua Fteah, a cave site in Libya [9,12]. Domesticated sheep probably moved westwards across the Mediterranean coast of northern Africa, down the Nile Valley, and across the then-wetter Sahara. They reached the Turkana basin by 3000 BCE [13,14].

When sheep first arrived in southernmost Africa has long been a source of significant contention [15,16,17,18,19,20,21,22,23,24,25,26,27]. Initially, the radiocarbon determinations used to date the arrival of sheep were on charcoal stratigraphically associated with sheep bones, and it was not clear how tight the association was between the charcoal dates and the bones. Indeed, direct dating on sheep remains from southern Africa showed that they must have migrated down through the sediments over time, as they were shown to be younger than the sediments from which they had been excavated [28,29]. The controversy appeared resolved with the direct radiocarbon dating of sheep bones. It later became clear that the morphological characteristics used to identify the bones of domesticated animals and distinguish them from the bones of the many wild bovids found in southern Africa are not reliable. Ancient DNA recovered from bones morphologically identified as domestic stock, or potentially as those of domestic stock, in South Africa and Lesotho have often revealed wild origins [30,31,32,33,34,35,36]. It should be noted that these results have been challenged on morphological grounds [37,38,39] and those challenges are disputed [40,41]. Protein analyses have likewise both revised [42] and confirmed [43] original morphological identifications as domestic stock in South Africa and Namibia. To the best of our knowledge, the protein-based correction of a morphological species assignment has not been challenged.

The relationship between southern Africa’s archaeological domesticated sheep and its modern domesticated sheep is not straightforward. Indigenous Khoekhoen began trading with European explorers in 1497 [44,45,46] and in 1652, the Dutch East India Company (Verenigde Oostindische Compagnie, VOC) established a provisioning station to supply company ships rounding the Cape of Good Hope. Initially, the provisioning station sourced its meat by trading with local hunters and herders, but by 1657 employees of the VOC had been established as free burghers (from the Dutch *burger*, or citizen) tasked with producing food to be sold to the VOC. Soon the burghers were managing successful farms and raising livestock on lands that extended well beyond those originally granted to them by the VOC [47].

In 1787 O.F. Mentzel published *A Geographical and Topographical Description of the Cape of Good Hope* in German. It was republished in English by The Van Riebeeck Society, Cape Town in 1921. In it, Mentzel describes sheep being imported to southernmost Africa from Persia (Iran) and England [48]. While no dates are provided for the importation of Persian sheep, Mentzel notes that Governor Willem Adriaan van der Stel managed to circumvent English laws preventing the exportation of breeding sheep and acquire some for his own farm [48] which he bred with Persian sheep being raised by the local free burghers. Van der Stel was the Governor of the Cape Colony from 1699 until 1707. Persian sheep had therefore been introduced to the Cape by 1699 at the latest, and English sheep sometime before 1707. While in the Cape Colony, van der Stel is reported to have carefully prevented interbreeding between his flock and the indigenous sheep managed by local Khoekhoen. Van der Stel was returned to the Netherlands in some disgrace in 1707 [49], after which, sheep of diverse origins interbred extensively [48].

**Figure 1:**
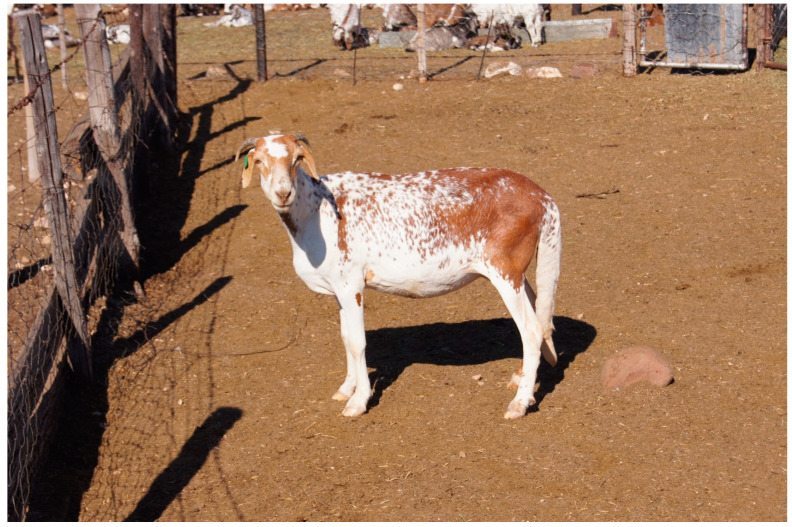
Damara sheep, specimen DAM25 (photograph credit: KAH).

The sheep breeds studied herein are the Damara, Namaqua Afrikaner, and Ronderib Afrikaner. These breeds are generally regarded as indigenous to southern Africa [50,51,52,53], but southern Africa’s long colonial history combined with the historical records of the importation of Persian and English sheep suggests that they are more likely of hybrid origin. All three of these breeds have several notable characteristics (see Figure 1). They have coarse hair, rather than wool, and a significant fatty deposit at the base of the tail and are regarded as drought tolerant and hardy [6,50,51,54,55,56,57].

Mitochondrial diversity in domesticated sheep clusters in to five haplogroups designated A–E [58,59,60,61,62,63,64,65,66,67,68]. Haplogroups A and B exhibit almost worldwide distribution, with haplogroup A being found at particularly high frequencies on the Indian subcontinent and haplogroup B at high frequency in Europe [69]. Haplogroup C has a much more restricted distribution, being found primarily in southwestern Asia, northern China, and Mongolia. Members of haplogroups D and E are relatively rare and have so far been found only in southwestern Asia [69]. Within Africa, haplogroup B is the most common lineage reported in Algeria [70], Egypt [71,72], Ethiopia [73,74], Kenya [75], Morocco [76], and South Africa [32,77]. All southern African sheep so far reported are members of haplogroup B, but short fragment recovery has prevented their assignment to subhaplogroups.

## 2. Materials and Methods

### 2.1. Modern Samples

**Figure 2:**
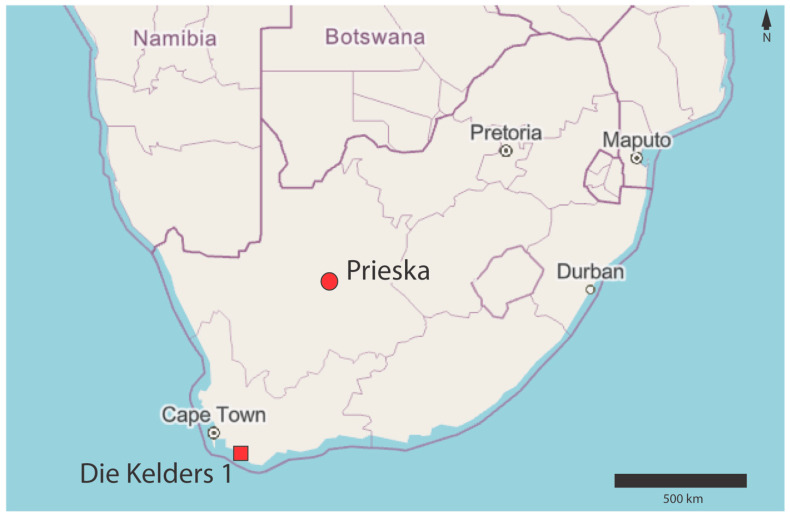
Map of southern Africa showing the locations of Prieska, Northern Cape, where modern sheep were sampled, and Die Kelders 1, the archaeological site from which the ancient specimens were excavated.

The sheep analyzed in this study were members of a flock farmed by Dawie du Toit at his Damara Stud Farm in Prieska, Northern Cape, South Africa (see Figure 2). De Toit took ear snips from 31 Damara, 15 Namaqua Afrikaner, and 9 Ronderib Afrikaner sheep. Ear snips were stored in 90% ethanol at room temperature while in the field, and then at −4 °C once they arrived at the laboratory. DNA was extracted using the DNeasy Blood & Tissue kit (QIAGEN, Hilden, Germany) following the manufacturer’s protocol, with a small modification. The kit specifies up to 8 h of incubation at 56 °C with ATL buffer and proteinase K. We found that 8 h of incubation was insufficient and extended incubation times to 12–18 h. Following incubation, hair and cartilage were centrifuged for collection and the lysate was transferred to a new tube before proceeding with the protocol.

Total DNA concentration was measured by spectrophotometry using a NanoDrop 2000 (Thermo Fisher Scientific, Waltham, MA, USA). Approximately 500 ng of DNA was used to construct barcoded Illumina sequencing libraries using the NEBNext Ultra II DNA Library Prep Kit for Illumina and the NEBNext Multiplex Oligos for Illumina (New England BioLabs, Ltd., Ipswich, MA, USA) following the manufacturer’s protocol. Barcoded libraries were quantified using the NEBNext Library Quant Kit for Illumina on the Bio-Rad CFX96 Touch Real-Time PCR Detection System. Libraries were pooled in equimolar ratios and sequenced on one PE150 S2 NovaSeq6000 Flowcell (Maryland Genomics, Baltimore, MD, USA).

### 2.2. Archaeological Samples

In an attempt to extract additional information from already analyzed archaeological sheep specimens without undertaking further destructive analyses, we re-extracted DNA from twelve freezer-stored bone digests from earlier work (see Table 1 for specimen details, [77]). All specimens were excavated from the deeply stratified cave site, Die Kelders 1, located approximately 120 km southeast of Cape Town (see Figure 1). The sheep analyzed here were excavated from Layer 2 of the Later Stone Age deposits [78,79,80,81,82,83] which date to AD 630–857 (SHCal13 Curve, [84,85]). Specimens were selected to ensure that the same individual was not sampled twice. Most of the specimens are right half-mandibles and in the few cases in which left half-mandibles were selected, their morphology was examined by an expert zooarchaeologist (R. Klein, Stanford University) to ensure that they could not have derived from the same individual as the right half-mandibles. DNA extraction and the preparation of Illumina sequencing libraries were performed in the ancient DNA lab of the Southern Methodist University Molecular Anthropology Laboratories. DNA was extracted following Allentoft et al. [86], with only minor modifications of the protocol. Samples were not pre-digested with 5 mL of 0.5 M EDTA for 15 min to remove surface contamination because samples had been previously extracted. We were unable to achieve a 5 M solution of sodium acetate and substituted a 3 M solution.

Extracted DNA was used to construct double-barcoded Illumina sequencing libraries using the SRSLY PicoPlus Kit (Claret Bioscience, Santa Cruz, CA, USA). The molecules in each library were quantified using the NEBNext Library Quant Kit for Illumina on the Bio-Rad CFX96 Touch Real-Time PCR Detection System. These amplified libraries were visualized under UV with ethidium bromide on 2% agarose gels. Libraries were visually assessed for the presence of a spread of DNA fragments. Libraries with evidence of only adapters and primer dimers were not processed further.

MyBaits probes (MYcroarray, Ann Arbor, MI, USA) for sheep were used to enrich the sequencing libraries for the mitochondrial genome following the manufacturer’s protocol to low quantity and quality targets. The libraries were quantified using the NEBNext Library Quant Kit for Illumina on the Bio-Rad CFX96 Touch Real-Time PCR Detection System, pooled in equimolar ratios, and sequenced on a PE150 S2 NovaSeq6000 Flowcell (Maryland Genomics).

### 2.3. Data Analysis

For the modern samples, paired end sequence reads in a FASTQ format were aligned to a complete *Ovis aries* mitochondrial genome (Genbank accession: AF010406.1) using the maximal exact matches (MEM) command of the Burrow’s Wheeler Aligner (BWA) (Version 0.7.17) [87]. PCR duplicates were removed from the resulting BAM files using Picard’s MarkDuplicates (V.2.26) [88]. For the ancient specimens, the raw FASTQ files were first processed using AdapterRemoval2 (Version 2.3.2) [89]. This tool removed short reads (<25 bp), removed stretches of Ns and bases that had low-quality scores (<30), and finally merged the paired-end sequence reads which had an overlap of at least 11 nucleotides. These collapsed reads were then aligned to the complete *Ovis aries* mitochondrial genome (Genbank accession: AF010406.1) using the BWA aln command [87] using settings recommended for ancient DNA (specifically, seeding was disabled (−l 1014), the number of gap opens was set to 2 (−o 2) and the maximum edit distance was set to 0.03 (−n 0.03)). PCR duplicates were removed from the resulting BAM files using DeDup, a tool that has been specifically designed for ancient DNA reads. MapDamage (Version 2.0.2) was used to assess damage signatures associated with ancient DNA, specifically the transitions from C to T and G to A at the ends of the sequencing reads.

After these initial alignment steps, the ancient and modern samples were treated identically. Read groups were added using Picard’s AddOrReplaceReadGroups and Samtools was then used to exclude reads not mapping to the *Ovis aries* reference genome. Variant calling was performed using the GATK (Version 4.2.3) HaplotypeCaller. An in-house script was used to mask regions of coverage regions (minimum coverage 5×) before the GATK FastaAlternateReferenceMaker was used to generate FASTA sequences for downstream analyses. Additional *Ovis aries* complete mitochondrial genome were downloaded from Genbank (see Supplementary Materials Table S1) and aligned with the sequences from samples that had 100% DNA coverage using Mafft (Version 7). SeqMagick was used to generate the PHYLIP format files for uploading to PhyML 3.0 [90] (http://www.atgc-montpellier.fr/phyml/, accessed on 12 December 2021) to generate Maximum Likelihood phylogenetic trees. Smart Model Selection using the Akaike Information Criterion (AIC) was used [91] and the R package PopGenome was used to calculate genetic diversity statistics. 

## 3. Results

**Figure 3:**
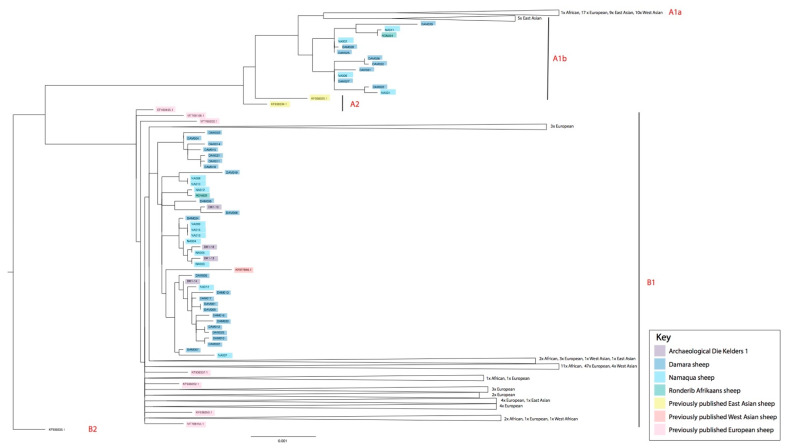
Phylogenetic tree showing sheep from the current study and comparative specimens, showing subhaplogroups A1a, A1b, A2, B1 and B2 (details listed in Supplementary Materials Table S1).

Complete, or almost complete, mitochondrial genomes were recovered from six of the archaeological specimens. The archaeological sheep are all members of subhaplogroup B1. Previous work on the same assemblage [77] also found the Die Kelders sheep to be members of haplogroup B, but with only 506 bp of sequence, that study was unable to determine subhaplogroup membership. Supplementary Materials Table S1 provides a complete list of specimens, percentage of the genome covered, the depth of coverage, and haplogroup assignments; Supplementary Materials Figure S1 shows the damage patterns indicative of ancient DNA. The supplementary materials of Lv et al. [69] list the mutations that define membership in each haplogroup and subhaplogroup. The modern sheep are dominated by subhaplogroup B1 (*n* = 42) but also include A1b (*n* = 13), previously unreported in southern African sheep. Supplementary Materials Figure S2 shows a phylogenetic tree including all the specimens analyzed here and previously geographically diverse representatives of haplogroups A and B; Supplementary Materials Figure S3 shows the network. The GenBank accession numbers, subhaplogroup assignments, geographical origin, and citations for all included specimens can be found on sheet 4 of Supplementary Materials Table S1. Figure 3 shows the tree with collapsed clades displaying the phylogenetic relationships.

As can be seen on the phylogenetic tree, the southern African sheep of the A1b lineage include members of all the sampled breeds—Damara, Namaqua Afrikaner, and Ronderib Afrikaner. European sheep that are members of haplogroup A cluster separately from the African haplogroup A sheep, suggesting that these A lineage sheep in southern Africa are not the direct and recent descendants of historically imported European sheep. Subhaplogroup A1b sheep are found in east Asia, but as can be seen on the phylogeny, the South African A1b sheep form a cluster separate from those of east Asia. It is therefore likely that these southern African sheep are not the recent descendants of imports from east Asia. None of the ancient specimens, however, are members of subhaplogroup A1b. Interestingly, these sheep possess fat-tails and are members of either haplogroup A or B, but outside of Africa, haplogroup C has been found to possess the highest frequencies of sheep with the fat-tail phenotype [69].

All the archaeological specimens and the majority of the modern ones are members of subhaplogroup B1. Interestingly, many of the modern B1 individuals are very closely related to the ancient B1 sheep, likely signaling that those modern B1 sheep share an ancestor-descendant relationship with the ancient sheep from Die Kelders 1.

Supplementary Materials Table S1, sheet 3 shows nucleotide diversity and haplotype diversity statistics for the sheep in this study and the comparative cattle and sheep data discussed here. Consistent with the patterns found in cattle in southern Africa [92,93], southern African sheep show less genetic diversity than sheep further north on the continent. There is approximately an order of magnitude lower within-haplogroup nucleotide diversity in the southern African sheep than has been reported in ancient and modern Turkish sheep [4]. Presumably, serial founder effects as populations moved south from their northerly origins resulted in a reduction of genetic diversity. While it is very unlikely that the same archaeological sheep were sampled twice, it is possible that the Die Kelders 1 sheep are very recently descended from the same maternal lineage.

## 4. Discussion

The genetic diversity of southern African sheep studied so far is markedly low compared with other sheep populations. Sheep took approximately 7000 years to move from northern to southern Africa, and in doing so, encountered a wide variety of environments from Mediterranean North Africa, across the equatorial regions, to subtropical southern Africa. The low level of genetic diversity in southern African sheep is consistent with models of serial founder effects as sheep populations moved across these diverse ecosystems.

Historical records document the importation of sheep to southernmost Africa from England and Persia (now Iran) during the late 17th and early 18th centuries [48]. Curiously, the sheep we have sequenced here show no evidence of these introductions of exotic sheep. The modern B lineage sheep are quite closely related to each other, not widely distributed through the B lineage clade, and many are especially closely related to the archaeological B lineage sheep. The deposits from which the archaeological sheep were excavated date to the middle of the first millennium AD, many hundreds of years before the first European explorers arrived in southern Africa. It seems likely that the modern B lineage sheep are descendants of some of the original southern African sheep.

The A lineage sheep among the modern specimens are phylogenetically distinct from the A lineage sheep so far reported from Europe and eastern Asia. This pattern suggests that these sheep are descendants of early southern African sheep as well, and not those introduced during the historic period. No A lineage sheep have been found among sequenced archaeological sheep from southern Africa, but given the small sample sizes studied so far, the pattern is more likely a consequence of small sample sizes than a genuine absence of A lineage sheep in prehistoric southern Africa. To date, sequenced archaeological sheep include only 20 individuals from Die Kelders 1 in the western Cape [77] and 1 from Blydefontein in the Karoo [32,41]. With such small samples, it is very likely that we have not yet captured an accurate picture of precontact diversity.

## 5. Conclusions

Little is known about the mitochondrial diversity of southern African sheep. Herein, we have made some progress towards addressing this gap in knowledge by sequencing the complete mitochondrial genomes of sheep from three indigenous southern African breeds, as well as a small sample of archaeological sheep. Close phylogenetic clustering of the archaeological sheep with some of the modern sheep suggests that many of southern Africa’s modern sheep are the direct descendants of the region’s prehistoric sheep. Further, those modern sheep that do not show particularly close phylogenetic relationships with the archaeological sheep cluster closely together, to the exclusion of European and Asian sheep. This pattern suggests that a larger sampling program of both archaeological and modern individuals will reveal additional diversity in southern African sheep across both time and space.

## Figures and Tables

**Table 1:** Archaeological specimens from Die Kelders 1.

Lab Identification	Element	Morphology	Provenience
DK1_02	Right mandible	P2 P3 P4 M1 M2 M3	Layer 2, AA2
DK1_04	Right mandible	M1 M2	Layer 2b, B3
DK1_05	Right mandible	dp3 dp4 M1 M2	Layer 2, A97
DK1_06	Right mandible	P2 M1 M2 M3	Layer 2, A2
DK1_09	Right mandible	dp2 dp3 dp4 M1	Layer 2, A3
DK1_10	Right mandible	M1 M2	Layer 2, A98
DK1_13	Right mandible	P3 P4	Layer 2, AA1
DK1_14	Right mandible	dp2 dp3 dp4	Layer 2, D2
DK1_16	Left mandible	dp2 dp4 M1	Layer 2, A98
DK1_25	Right mandible	dp3 dp4	Layer 2, A9b
DK1_53	Left mandible	dp2 dp3 dp4	Layer 2, B3
DK1_54	Left mandible	P2 P3 P4 M1 M2 M3	Layer 2, B3

## Data Availability

All DNA sequences are available in GenBank (accession numbers: pending).

## Supplementary Materials

The following are available online at https://www.mdpi.com/article/10.3390/biology11030428/s1, Figure S1: Phylogenetic tree of analyzed specimens and GenBank derived comparative specimens, Figure S2: Phylogenetic tree of analyzed specimens and diverse geographic representatives of haplogroups A and B. Figure S3: Maximum likelihood network of analyzed specimens and diverse geographic representatives of haplogroups A and B. Table S1: Individualized haplogroup assignments, diversity statistics, and details for the comparative sequences.

## References

[1] Kosgey I., Rowlands G., van Arendonk J., Baker R. (2008). Small ruminant production in smallholder and pastoral/extensive farming systems in Kenya. Small Rumin. Res..

[2] Legge T., Harris D.R. (1996). The beginning of caprine domestication in Southwest Asia. The Origins and Spread of Agriculture and Pastoralism in Eurasia.

[3] Álvarez I., Capote J., Traoré A., Fonseca N., Pérez K., Cuervo M., Fernández I., Goyache F. (2013). Mitochondrial analysis sheds light on the origin of hair sheep. Anim. Genet..

[4] Demirci S., Baştanlar E.K., Dagtas N., Pişkin E., Engin A., Ozer F., Yüncü E., Dogan S.A., Togan I. (2013). Mitochondrial DNA diversity of modern, ancient and wild sheep (*Ovis gmelinii anatolica*) from Turkey: New insights on the evolutionary history of sheep. PLoS ONE.

[5] Finlay E., Tapio M., Kierstein G., Jianlin H., Hanotte O. (2007). Assessment of Sheep Domestication History Based on Mitochondrial DNA Diversity of Contemporary Old World Sheep.

[6] Muigai A.W.T., Hanotte O. (2013). The origin of African sheep: Archaeological and genetic perspectives. Afr. Archaeol. Rev..

[7] di Lernia S., di Lernia S., Manzi G. (1998). Cultural control over wild animals during the early Holocene: The case of Barbary sheep in the central Sahara. Before Food Production in North Africa.

[8] di Lernia S. (1999). The Uan Afuda Cave: Hunter-Gatherer Societies of the Central Sahara (Arid Zone Archaeology Monographs).

[9] Klein R.G., Scott K. (1986). Re-analysis of faunal assemblages from the Haua Fteah and other late quaternary archaeological sites in Cyrenaican Libya. J. Archaeol. Sci..

[10] Saxon E.C., Close A.E., Cluzel C., Morse V., Shackleton N.J. (1974). Results of recent investigations at Tamar Hat. Libyca.

[11] Manwell C., Baker C.M.A. (1975). *Ammotragus lervia*: Pregenitor of domesticated sheep or specialized offshoot of caprine evolution. Experientia.

[12] Barker G., Basell L., Brooks I., Burn L., Cartwright C., Cole F., Davison J., Farr L., Grün R., Hamilton R. (2008). The Cyrenaican Prehistory Project 2008: The second season of investigations of the Haua Fteah cave and its landscape, and further results from the initial (2007) fieldwork. Libyan Stud..

[13] Barthelme J.W. (1985). Fisher-Hunters and Neolithic Pastoralists in East Turkana, Kenya.

[14] Culley C., Janzen A., Brown S., Prendergast M.E., Shipton C., Ndiema E., Petraglia M.D., Boivin N., Crowther A. (2021). Iron Age hunting and herding in coastal eastern Africa: ZooMS identification of domesticates and wild bovids at Panga ya Saidi, Kenya. J. Archaeol. Sci..

[15] Sadr K. (1998). The first herders at the Cape of Good Hope. Afr. Archaeol. Rev..

[16] Sadr K. (2003). The Neolithic of southern Africa. J. Afr. Hist..

[17] Sadr K. (2015). Livestock first reached southern Africa in two separate events. PLoS ONE.

[18] Smith A.B. (1983). Prehistoric pastoralism in the Southwestern Cape, South Africa. World Archaeol..

[19] Smith A.B. (1987). The Economics of Prehistoric Herding at the Cape. Final Report to the Human Sciences Research Council.

[20] Smith A.B. (1992). Origins and spread of pastoralism in Africa. Annu. Rev. Anthropol..

[21] Smith A.B. (1998). Early domestic stock in southern Africa: A commentary. Afr. Archaeol. Rev..

[22] Smith A.B., Blench R.M., MacDonald K.C. (2000). The origins of the domesticated animals of southern Africa. The Origins and Development of African Livestock: Archaeology, Genetics, Linguistics and Ethnography.

[23] Smith A.B. (2008). Pastoral origins at the Cape, South Africa: Influences and arguments. South. Afr. Humanit..

[24] Lander F., Russell T. (2018). The archaeological evidence for the appearance of pastoralism and farming in southern Africa. PLoS ONE.

[25] Russell T. (2017). “Where goats connect people”: Cultural diffusion of livestock not food production amongst southern African hunter-gatherers during the Later Stone Age. J. Soc. Archaeol..

[26] Russell T., Lander F. (2015). “What is consumed is wasted”: From foraging to herding in the southern African Later Stone Age. Azania Archaeol. Res. Afr..

[27] Russell T.M. (2004). The Spatial Analysis of Radiocarbon Databases: The Spread of the First Farmers in Europe and of the Fat-Tailed Sheep in Southern Africa.

[28] Sealy J., Yates R. (1994). The chronology of the introduction of pastoralism to the Cape, South Africa. Antiquity.

[29] Sealy J., Yates R. (1996). Direct radiocarbon dating of early sheep bones: Two further results. South Afr. Archaeol. Bull..

[30] Orton J., Mitchell P., Klein R., Steele T., Horsburgh K.A. (2013). An early date for cattle from Namaqualand, South Africa: Implications for the origins of herding in southern Africa. Antiquity.

[31] Horsburgh K.A. (2015). Molecular anthropology: The judicial use of genetic data in archaeology. J. Archaeol. Sci..

[32] Horsburgh K.A., Moreno-Mayar J.V. (2015). Molecular identification of sheep at Blydefontein Rock Shelter, South Africa. South. Afr. Humanit..

[33] Horsburgh K.A., Moreno-Mayar J.V., Gosling A.L. (2016). Revisiting the Kalahari debate in the highlands: Ancient DNA provides new faunal identifications at Sehonghong, Lesotho. Azania Archaeol. Res. Afr..

[34] Horsburgh K.A., Orton J., Klein R.G. (2016). Beware the Springbok in sheep’s clothing: How secure are the faunal identifications upon which we build our models?. Afr. Archaeol. Rev..

[35] Horsburgh K.A. (2020). Genetics and domestic fauna in southern Africa. Oxford Research Encyclopedia of Anthropology.

[36] Horsburgh K.A., Gosling A.L. (2020). Systematic ancient DNA species identification fails to find late Holocene domesticated cattle in southern Africa. Biology.

[37] Scott K., Plug I. (2016). Osteomorphology and osteometry versus aDNA in taxonomic identification of fragmentary sheep and sheep/goat bones from archaeological deposits: Blydefontein Shelter, Karoo, South Africa. South. Afr. Humanit..

[38] Plug I. (2017). Reply to Horsburgh et al. 2016: “Revisiting the Kalahari debate in the highlands”. Azania Archaeol. Res. Afr..

[39] Bousman B.C., Mauldin R., Zoppi U., Higham T., Scott L., Brink J. (2016). The quest for evidence of domestic stock at Blydefontein Rock Shelter. South. Afr. Humanit..

[40] Horsburgh K.A. (2017). A reply to Plug 2017: Science requires self-correction. Azania Archaeol. Res. Afr..

[41] Horsburgh K.A., Moreno-Mayar J.V., Klein R.G. (2017). Counting and miscounting sheep: Genetic evidence for pervasive misclassification of wild fauna as domestic stock. South. Afr. Humanit..

[42] Le Meillour L., Zirah S., Zazzo A., Cersoy S., Détroit F., Imalwa E., Lebon M., Nankela A., Tombret O., Pleurdeau D. (2020). Palaeoproteomics gives new insight into early southern African pastoralism. Sci. Rep..

[43] Coutu A.N., Taurozzi A.J., Mackie M., Jensen T.Z.T., Collins M.J., Sealy J. (2021). Palaeoproteomics confirm earliest domesticated sheep in southern Africa ca. 2000 BP. Sci. Rep..

[44] Raven-Hart R. (1967). Before Van Riebeeck: Callers at South Africa from 1488 to 1652.

[45] Heinrich A.R., Schrire C., Schablitsky J., Leone M. (2011). Colonial fauna at the Cape of Good Hope: A proxy for colonial impact on indigenous people. The Importance of Material Things.

[46] Schrire C. (2014). Historical Archaeology in South Africa: Material Culture of the Dutch East India Company at the Cape.

[47] Thompson L. (1995). A History of South Africa.

[48] Mentzel O.F., Mandelbrote H.J. (1921). A Geographical and Topographical Description of the Cape of Good Hope.

[49] Gordon R.E., Talbot C.J. (1977). From Dias to Vorster: Source Material on South African History 1488–1975.

[50] du Toit D. (2007). The Damara of southern Africa. Anim. Genet. Resour. Inf..

[51] Campbell Q.P. (2003). The origin and description of southern Africa’s indigenous goats. South Afr. J. Anim. Sci..

[52] Almeida A.M. (2011). The Damara in the context of southern Africa fat-tailed sheep breeds. Trop. Anim. Health Prod..

[53] Kilminster T.F., Greeff J.C. (2011). A note on the reproductive performance of Damara, Dorper and Merino sheep under optimum management and nutrition for Merino ewes in the eastern wheatbelt of western Australia. Trop. Anim. Health Prod..

[54] Epstein H., Epstein H. (1971). The fat-tailed sheep of Africa. The Origin of Domestic Animals of Africa.

[55] Epstein H. (1960). History and origin of the Ronderib and Namaqua Afrikaner sheep. Z. Tierzüchtung Züchtungsbiol..

[56] Thutwa K., van Wyk J.B., Dzama K., Scholtz A.J., Cloete S.W. (2021). Expression of cytokine genes at tick attachment and control sites of Namaqua Afrikaner, Dorper and South African Mutton Merino sheep. Vet. Parasitol..

[57] Moradi M.H., Nejati-Javaremi A., Moradi-Shahrbabak M., Dodds K.G., McEwan J.C. (2012). Genomic scan of selective sweeps in thin and fat tail sheep breeds for identifying of candidate regions associated with fat deposition. BMC Genet..

[58] Wood N.J., Phua S.H. (1996). Variation in the control region sequence of the sheep mitochondrial genome. Anim. Genet..

[59] Hiendleder S., Kaupe B., Wassmuth R., Janke A. (2002). Molecular analysis of wild and domestic sheep questions current nomenclature and provides evidence for domestication from two different subspecies. Proc. R. Soc. B Boil. Sci..

[60] Hiendleder S., Lewalski H., Wassmuth R., Janke A. (1998). The complete mitochondrial DNA sequence of the domestic sheep (*Ovis aries*) and comparison with the other major ovine haplotype. J. Mol. Evol..

[61] Hiendleder S., Mainz K., Plante Y., Lewalski H. (1998). Analysis of mitochondrial DNA indicates that domestic sheep are derived from two different ancestral maternal sources: No evidence for contributions from urial and argali sheep. J. Hered..

[62] Hiendleder S., Phua S.H., Hecht W. (1999). A diagnostic assay discriminating between two major *Ovis aries* mitochondrial DNA haplotypes. Anim. Genet..

[63] Meadows J.R.S., Hiendleder S., Kijas J.W. (2011). Haplogroup relationships between domestic and wild sheep resolved using a mitogenome panel. Heredity.

[64] Guo J., Du L.-X., Ma Y.-H., Guan W.-J., Li H.-B., Zhao Q.-J., Li X., Rao S.-Q. (2005). A novel maternal lineage revealed in sheep (*Ovis aries*). Anim. Genet..

[65] Pedrosa S., Arranz J.-J., Brito N., Molina A., Primitivo F.S., Bayón Y. (2007). Mitochondrial diversity and the origin of Iberian sheep. Genet. Sel. Evol..

[66] Pedrosa S., Uzun M., Arranz J.-J., Gutiérrez-Gil B., Primitivo F.S., Bayón Y. (2005). Evidence of three maternal lineages in near eastern sheep supporting multiple domestication events. Proc. R. Soc. B Boil. Sci..

[67] Meadows J., Li K., Kantanen J., Tapio M., Sipos W., Pardeshi V., Gupta V., Calvo J., Whan V., Norris B. (2005). Mitochondrial sequence reveals high levels of gene flow between breeds of domestic sheep from Asia and Europe. J. Hered..

[68] Tapio M., Marzanov N., Ozerov M., Ćinkulov M., Gonzarenko G., Kiselyova T., Murawski M., Viinalass H., Kantanen J. (2006). Sheep mitochondrial DNA variation in European, Caucasian, and central Asian areas. Mol. Biol. Evol..

[69] Lv F.-H., Peng W.-F., Yang J., Zhao Y.-X., Li W.-R., Liu M.-J., Ma Y.-H., Zhao Q.-J., Yang G.-L., Wang F. (2015). Mitogenomic meta-analysis identifies two phases of migration in the history of eastern Eurasian sheep. Mol. Biol. Evol..

[70] Ghernouti N., Bodinier M., Ranebi D., Maftah A., Petit D., Gaouar S. (2017). Control region of mtDNA identifies three migration events of sheep breeds in Algeria. Small Rumin. Res..

[71] Othman O.E.M., Pariset L., Balabel E.A., Marioti M. (2015). Genetic characterization of Egyptian and Italian sheep breeds using mitochondrial DNA. J. Genet. Eng. Biotechnol..

[72] Othman O.E., Balabel E.A., Abdel-Samad M.F. (2014). Mitochondrial DNA diversity in five Egyptian sheep breeds. Glob. Vet..

[73] Nigussie H., Mwacharo J.M., Osama S., Agaba M., Mekasha Y., Kebede K., Abegaz S., Pal S.K. (2020). Correction to: Genetic diversity and matrilineal genetic origin of fat-rumped sheep in Ethiopia. Trop. Anim. Health Prod..

[74] Nigussie H., Mwacharo J.M., Osama S., Agaba M., Mekasha Y., Kebede K., Abegaz S., Pal S.K. (2019). Genetic diversity and matrilineal genetic origin of fat-rumped sheep in Ethiopia. Trop. Anim. Health Prod..

[75] Resende A., Gonçalves J., Muigai A.W.T., Pereira F. (2016). Mitochondrial DNA variation of domestic sheep (*Ovis aries*) in Kenya. Anim. Genet..

[76] Kandoussi A., Boujenane I., Auger C., Serranito B., Germot A., Piro M., Maftah A., Badaoui B., Petit D. (2020). The origin of sheep settlement in western Mediterranean. Sci. Rep..

[77] Horsburgh K.A., Rhines A. (2010). Genetic characterization of an archaeological sheep assemblage from South Africa’s Western Cape. J. Archaeol. Sci..

[78] Schweitzer F.R. (1979). Excavations at Die Kelders, Cape Province, South Africa: The Holocene deposits. Ann. South Afr. Mus..

[79] Avery G., Cruz-Uribe K., Goldberg P., Grine F.E., Klein R.G., Lenardi M.J., Marean C.W., Rink W.J., Schwarcz H.P., Thackeray A.I. (1997). The 1992–1993 Excavations at the Die Kelders Middle and Later Stone Age Cave site, South Africa. J. Field Archaeol..

[80] Goldberg P. (2000). Micromorphology and site formation at Die Kelders Cave I., South Africa. J. Hum. Evol..

[81] Klein R.G., Cruz-Uribe K. (2000). Middle and Later Stone Age large mammal and tortoise remains from Die Kelders Cave 1, Western Cape Province, South Africa. J. Hum. Evol..

[82] Klein R.G., Cruz-Uribe K. (1996). Exploitation of large bovids and seals at Middle and Later Stone Age sites in South Africa. J. Hum. Evol..

[83] Wilson M.L. (1996). The late Holocene occupants of Die Kelders: Hunter-gatherers or herders?. South. Afr. Field Archaeol..

[84] Schweitzer F.R. (1974). Archaeological evidence for sheep at the Cape. South Afr. Archaeol. Bull..

[85] Hogg A.G., Hua Q., Blackwell P.G., Niu M., Buck C., Guilderson T.P., Heaton T.J., Palmer J., Reimer P.J., Reimer R.W. (2013). SHCal13 southern hemisphere calibration, 0–50,000 years cal BP. Radiocarbon.

[86] Allentoft M.E., Sikora M., Sjögren K.G., Rasmussen S., Rasmussen M., Stenderup J., Damgaard P.B., Schroeder H., Ahlström T., Vinner L. (2015). Population genomics of Bronze Age Eurasia. Nature.

[87] Li H., Durbin R. (2009). Fast and accurate short read alignment with Burrows–Wheeler transform. Bioinformatics.

[88] Jónsson H., Ginolhac A., Schubert M., Johnson P.L.F., Orlando L. (2013). mapDamage2.0: Fast approximate Bayesian estimates of ancient DNA damage parameters. Bioinformatics.

[89] Lindgreen S. (2012). AdapterRemoval: Easy cleaning of next-generation sequencing reads. BMC Res. Notes.

[90] Guindon S., Dufayard J.-F., Lefort V., Anisimova M., Hordijk W., Gascuel O. (2010). New algorithms and methods to estimate maximum-likelihood phylogenies: Assessing the performance of PhyML 3.0. Syst. Biol..

[91] Lefort V., Longueville J.-E., Gascuel O. (2007). SMS: Smart Model Selection in PhyML. Mol. Biol. Evol..

[92] Horsburgh K.A., Prost S., Gosling A., Stanton J.-A., Rand C., Matisoo-Smith E.A. (2013). The genetic diversity of the Nguni breed of African cattle (*Bos* spp.): Complete mitochondrial genomes of haplogroup T1. PLoS ONE.

[93] Bonfiglio S., Ginja C., De Gaetano A., Achilli A., Olivieri A., Colli L., Tesfaye K., Agha S.H., Gama L.T., Cattonaro F. (2012). Origin and spread of *Bos taurus*: New clues from mitochondrial genomes belonging to haplogroup T1. PLoS ONE.

