# Supplementary figures and images for: Maternal Relationships among Ancient and Modern Southern African Sheep: Newly Discovered Mitochondrial Haplogroups
Source: Biology (Basel). 2022 Mar 11;11(3):428. doi: 10.3390/biology11030428 (PMC8944976; doi:10.3390/biology11030428)

# DK1-10\_rdgtps

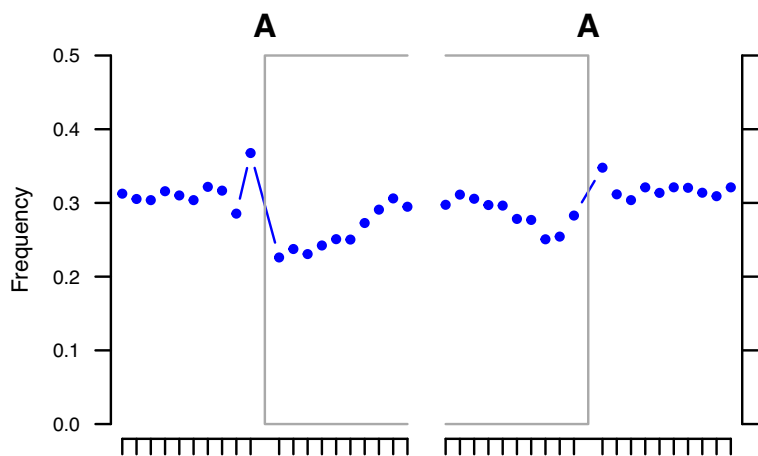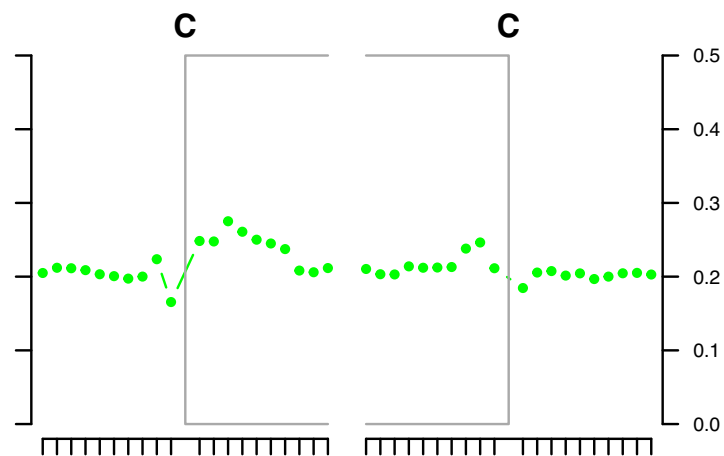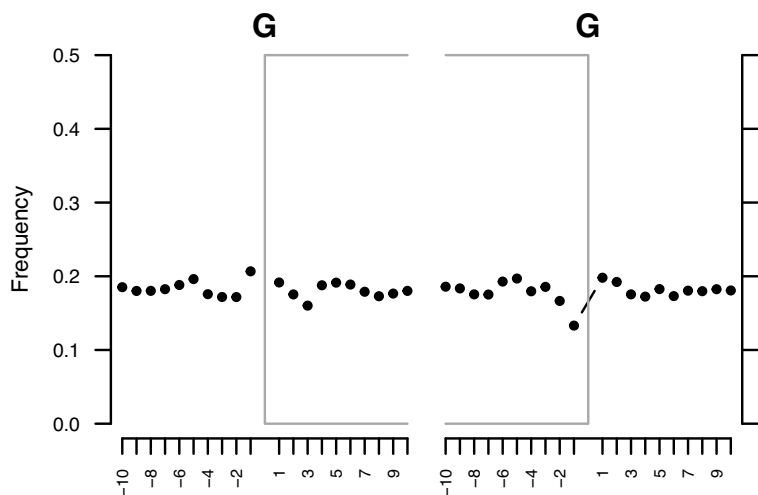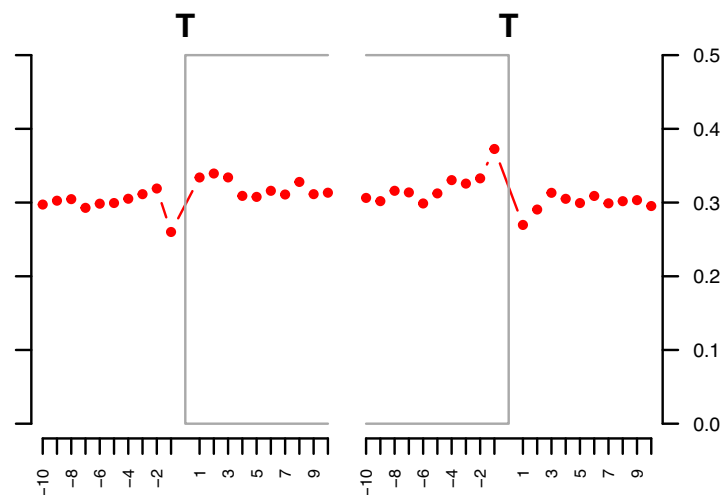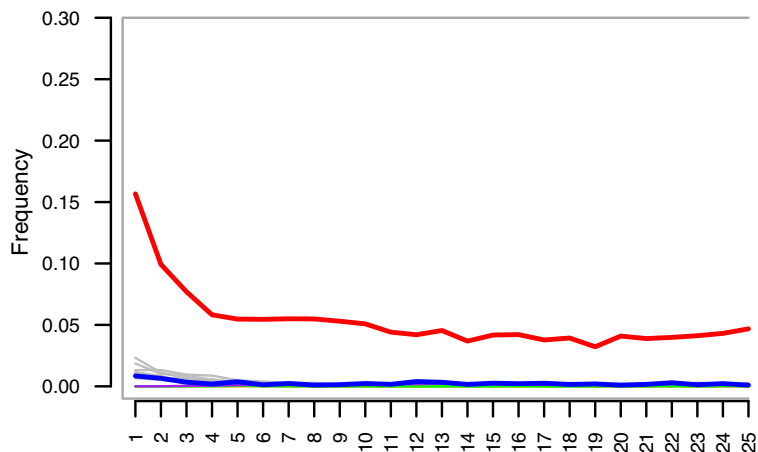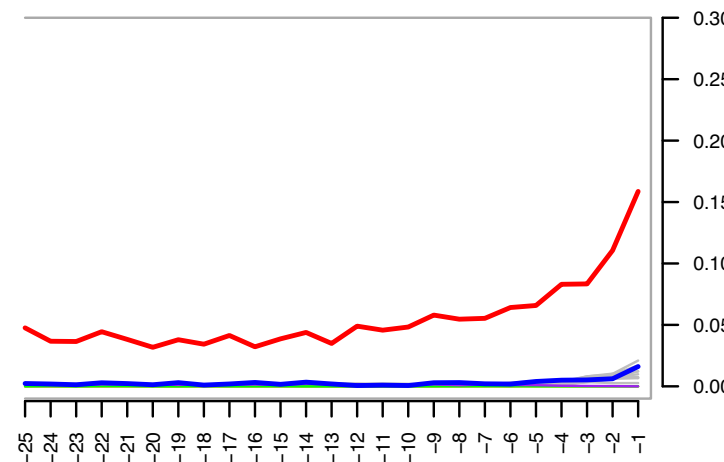

# DK1-13\_rdgtps

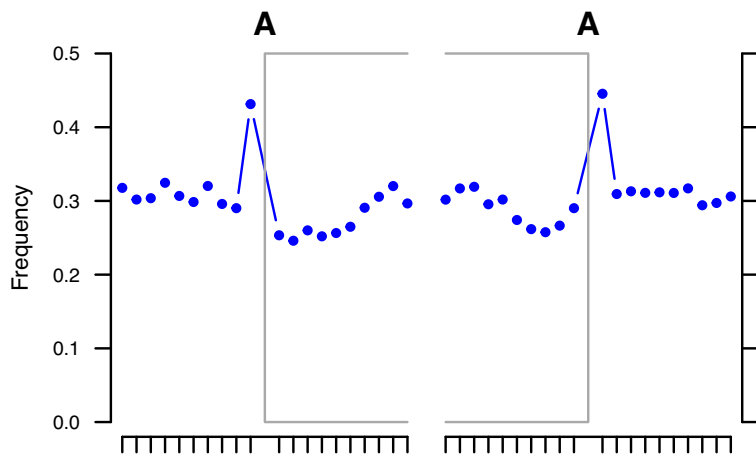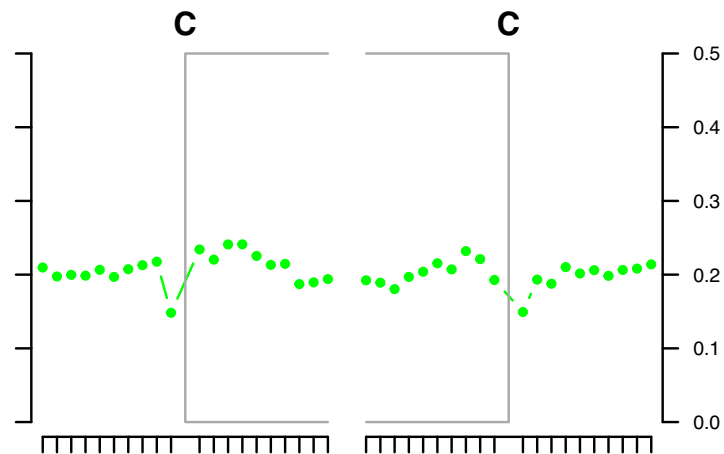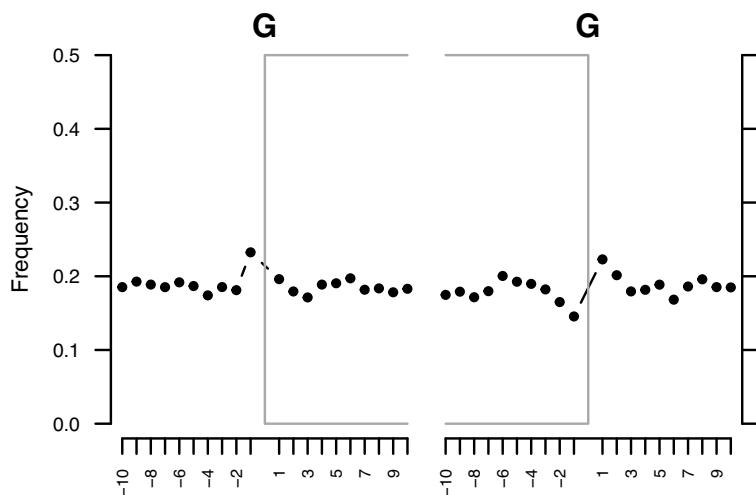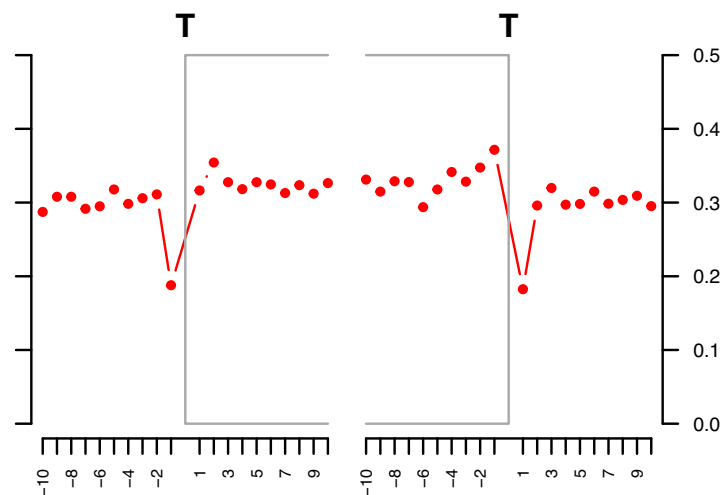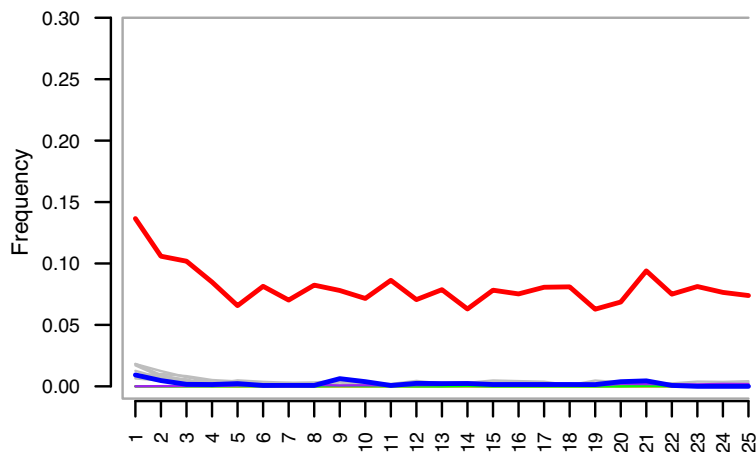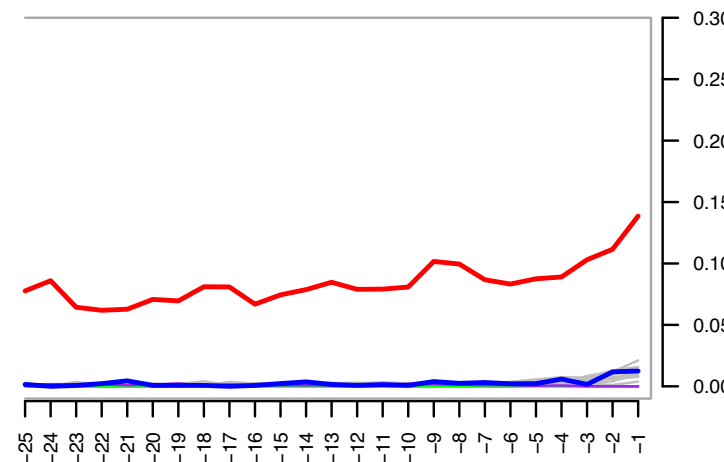

# DK1-14\_rdgtps

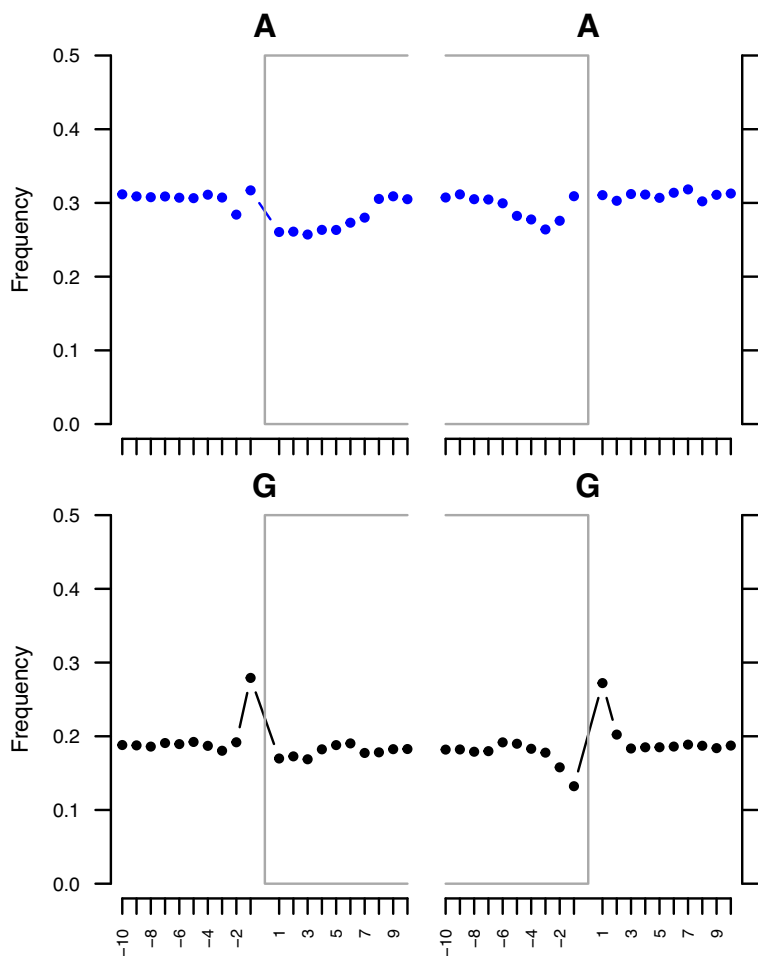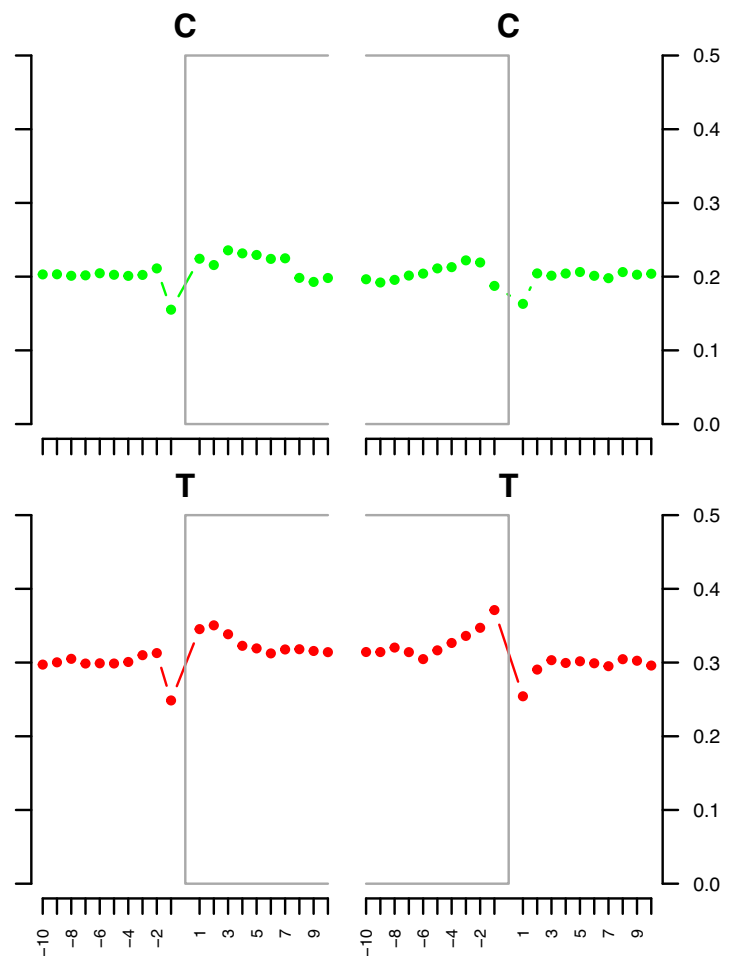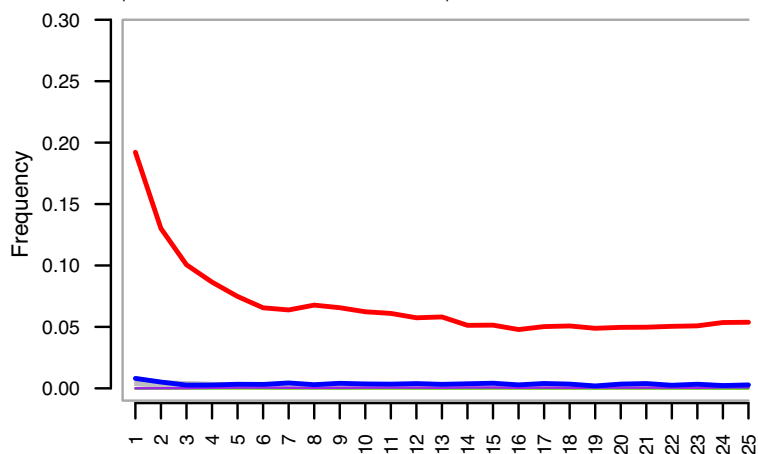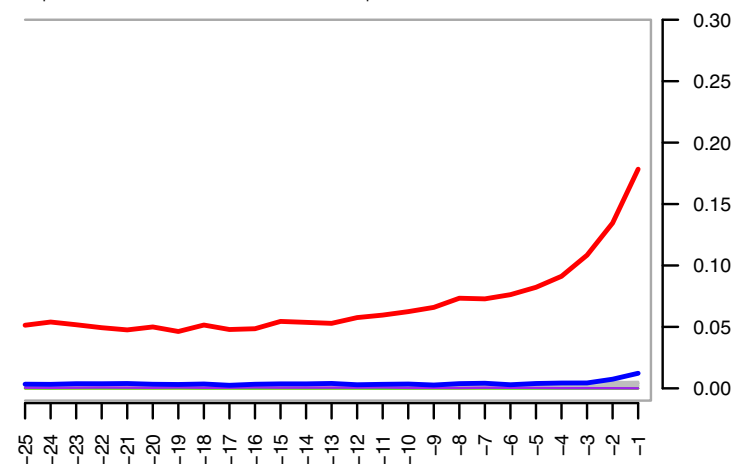

# DK1-16\_rdgtps

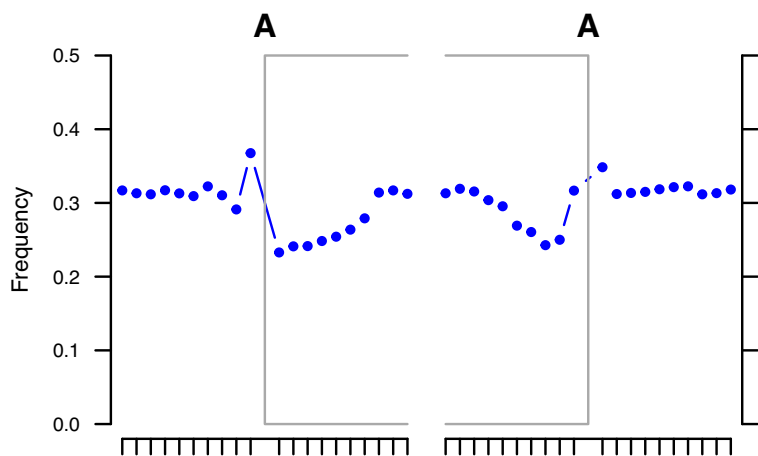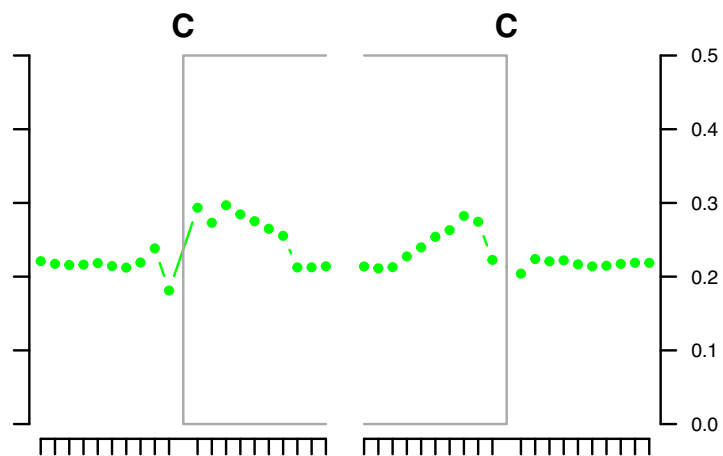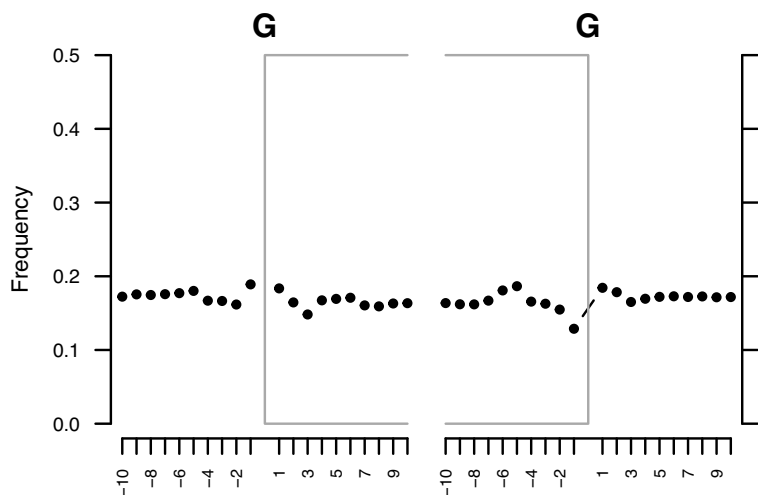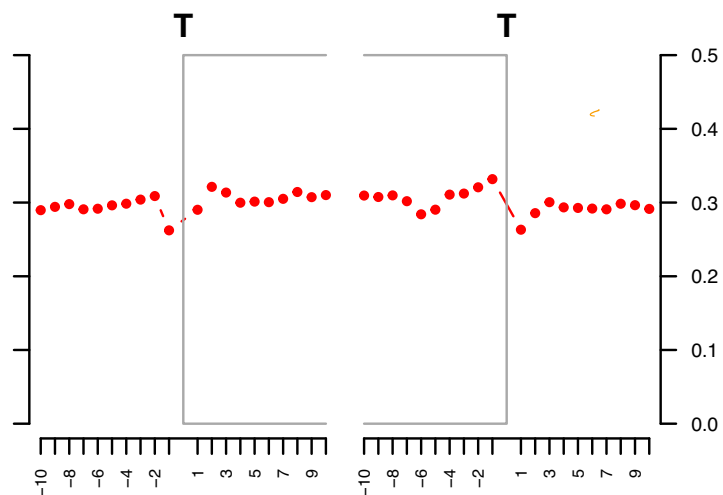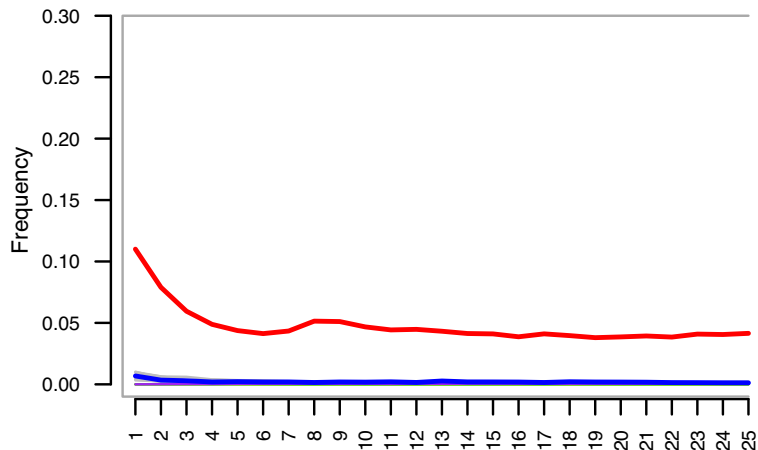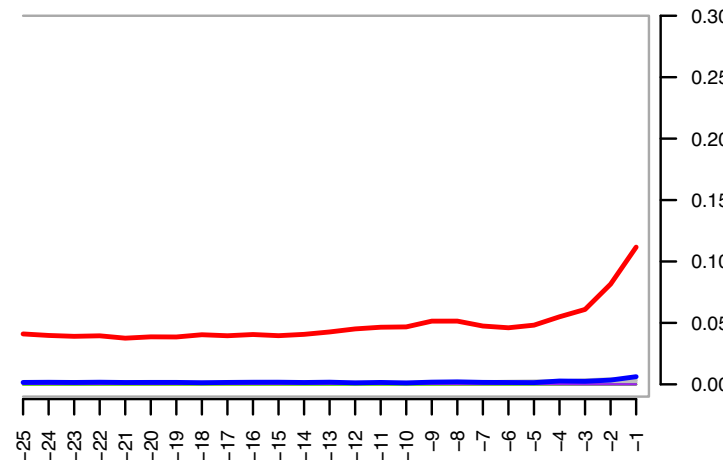

# DK1-54\_rdgtps

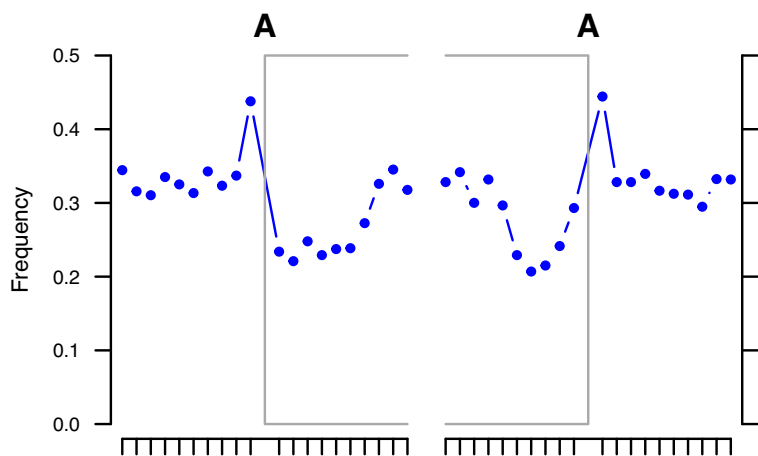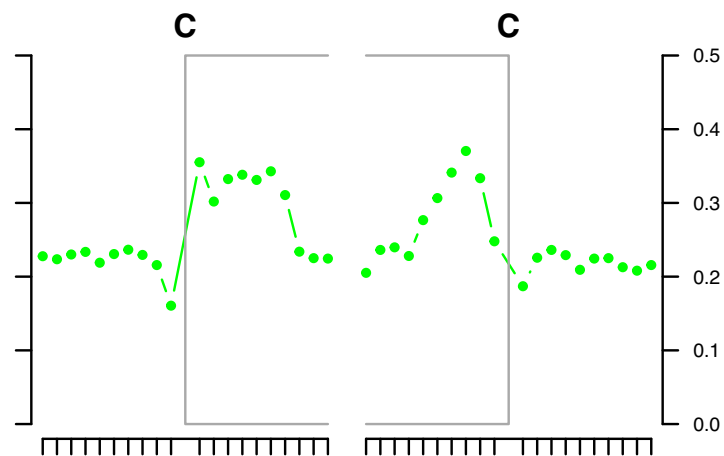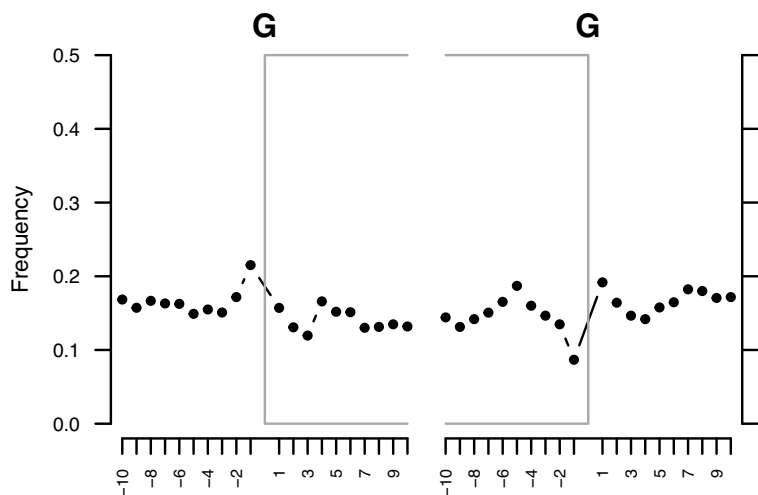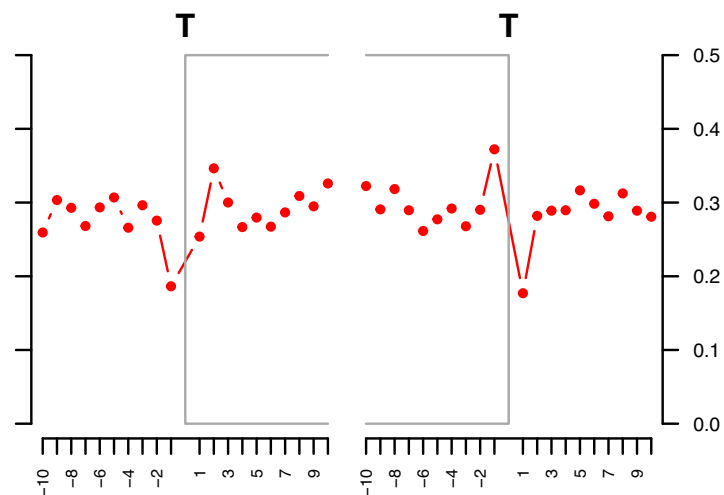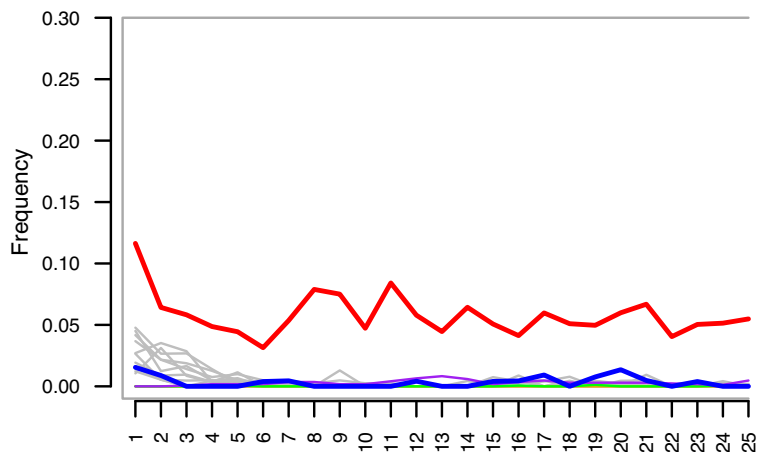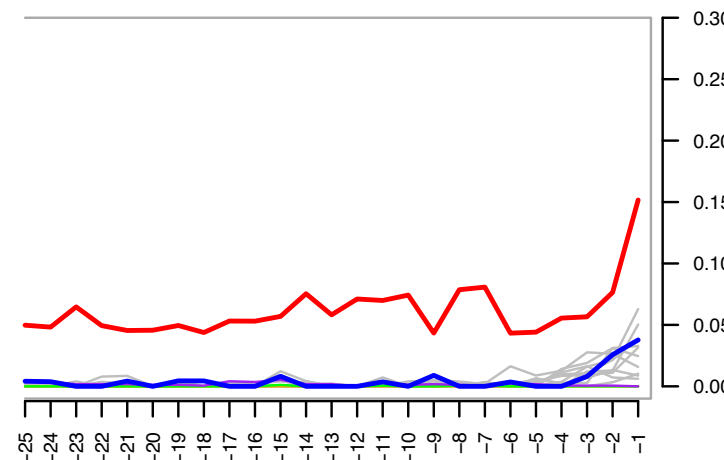

Supplement: Supplementary file 1 [file biology-11-00428-s001.zip › Supplementary Materials Figure S1.pdf]

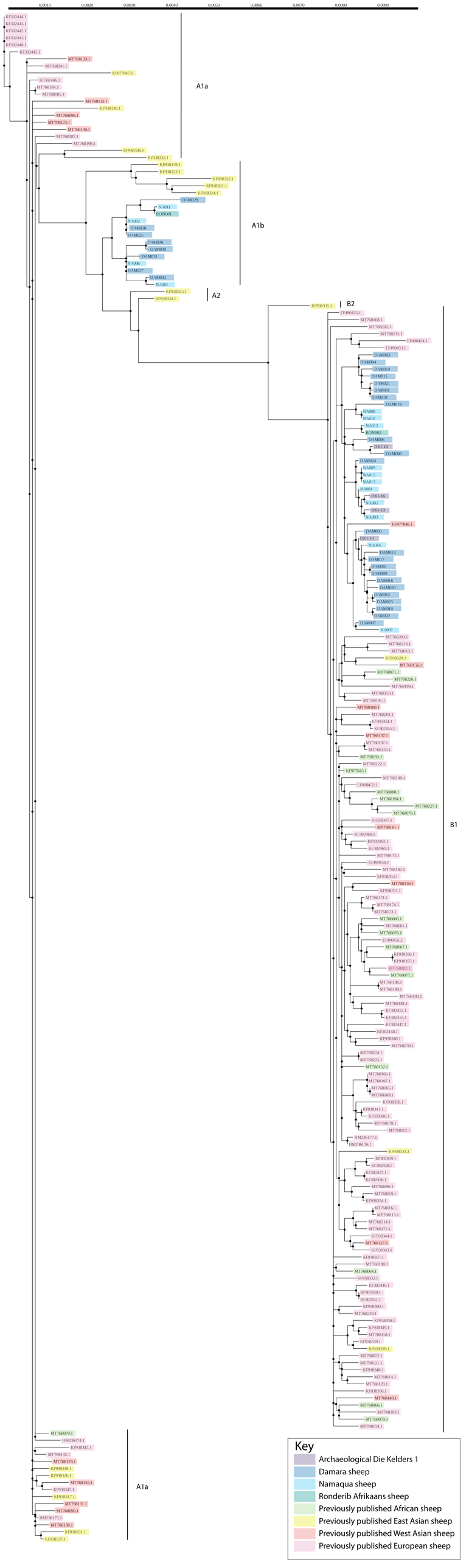

Supplement: Supplementary file 1 [file biology-11-00428-s001.zip › Supplementary Materials Figure S2.pdf]

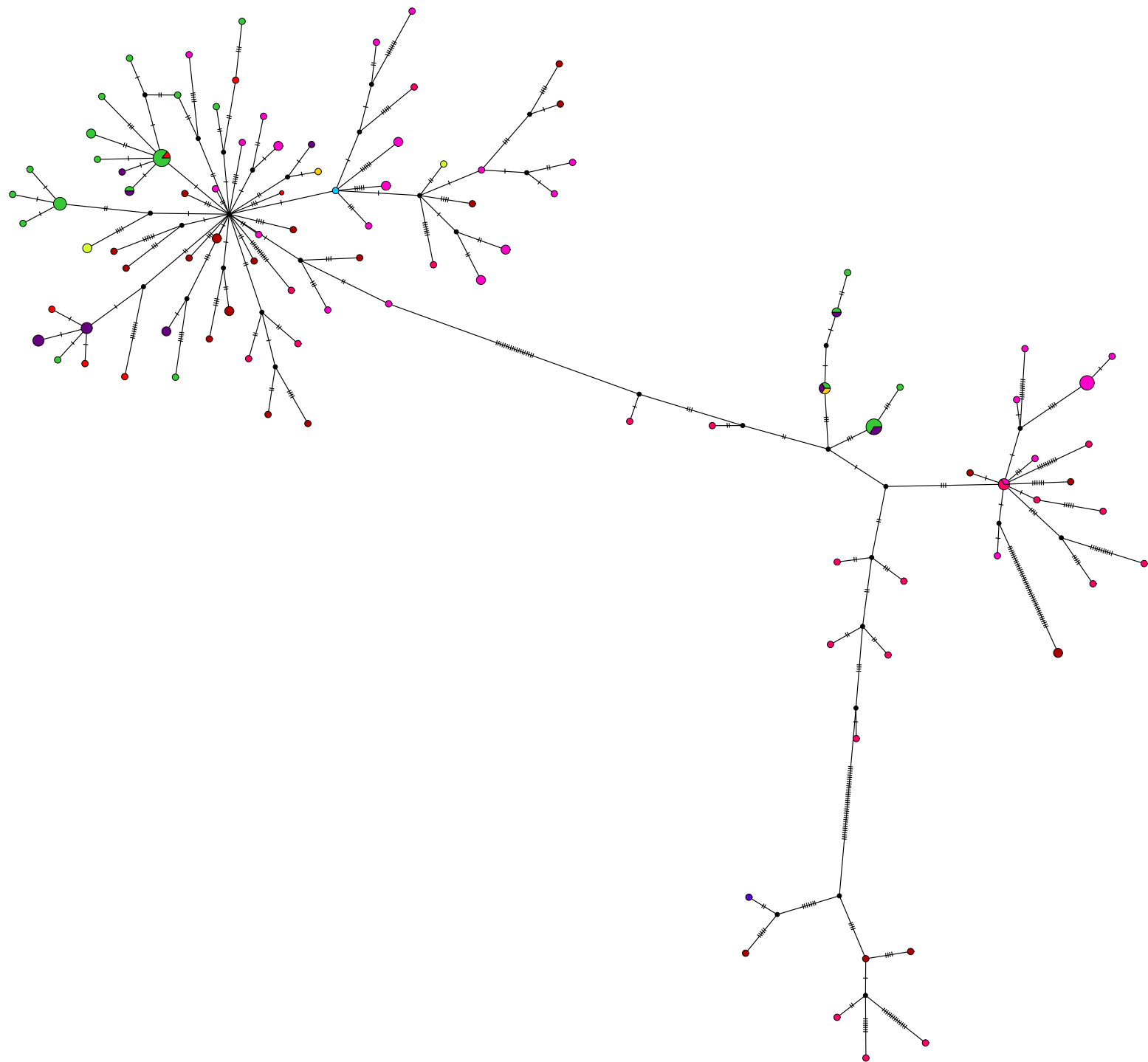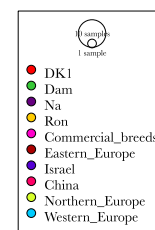

Supplement: Supplementary file 1 [file biology-11-00428-s001.zip › Supplementary Materials Figure S3.pdf]
